# Serological prevalence of six vector-borne pathogens in dogs presented for elective ovariohysterectomy or castration in the South central region of Texas

**DOI:** 10.1186/s12917-020-02607-w

**Published:** 2020-10-08

**Authors:** J. Mack Fudge, Bailey Boyanowski, Bernie Page, Shuling Liu, Artem S. Rogovskyy

**Affiliations:** 1 Hill Country Animal League, 924 N. Main St, Boerne, TX 78006 USA; 2 Statistical Collaboration Center, Department of Statistics, College of Science, Texas A&M University, College Station, TX 77843 USA; 3 Department of Veterinary Pathobiology, College of Veterinary Medicine and Biomedical Sciences, Texas A&M University, College Station, TX 77843 USA

**Keywords:** Anaplasmosis, Ehrlichiosis, Heartworm, Lyme borreliosis, Seroprevalence, Tick-borne pathogens

## Abstract

**Background:**

Most vector-borne pathogens cause zoonotic diseases. These zoonoses often have wild animal reservoirs that play a significant role in disease epidemiology. However, pet animals have also been implicated in transmission of zoonotic agents to humans. To exemplify, dogs are competent reservoir hosts for several zoonotic vector-borne bacteria and protozoa. Despite that vector-borne diseases can be life-threatening for both pets and humans, studies on pathogen seroprevalence are very limited. Therefore, the objective of this study was to determine the serological prevalence of six zoonotic vector-borne agents in dogs from the South Central region of Texas (US).

Electronic medical records of dogs, presenting over 2014–2019 for elective ovariohysterectomy or castration at a high volume spay and neuter clinic, were reviewed for serological testing. Sera from 418 dogs were tested for the *Dirofilaria immitis* antigen, and antibodies to *Anaplasma phagocytophilum, Anaplasma platys*, *Borrelia burgdorferi, Ehrlichia canis,* and *Ehrlichia ewingi,* using a commonly available commercial test kit. Descriptive statistics were computed to characterize the respective seroprevalence rates of the dog population. The study involved 192 (46%) male and 226 (54%) female dogs.

**Results:**

Overall, 85 (20%) dogs tested positive for at least one of the 6 pathogens investigated. The highest seroprevalence rate averaged over the 6-year period was 11.7% for *D. immitis* followed by 8.4% for *E. canis* and/or *E. ewingii*, 4.3% for *A. phagocytophilum* and/or *A. platys*, and 0.2% for *B. burgdorferi*. The co-exposure or co-infection was only detected in 3.8% of the dog population.

**Conclusions:**

Together, opportunistic testing of dogs presenting for elective surgical procedures may provide an effective way of assessing seroprevalence and/or risk factors for common vector-borne diseases within a geographic region of concern.

## Background

Vectors are commonly defined as blood-feeding arthropods (e.g., mosquitoes, ticks) that transmit pathogens between hosts [1, 2]. Unfortunately, diseases caused by the vector-borne pathogens are widespread world-wide. Reported human cases of vector-borne diseases in the United States (US) alone have more than tripled since 2004 and are overall characterized by steadily increasing incidence of tick-borne diseases and sporadic outbreaks of mosquito-borne diseases [3]. In the US, well over 600,000 human cases of vector-borne diseases were recorded during 2004–2016, with tick-borne diseases accounting for nearly three-quarters of those cases. In the same period, human cases of tick-borne diseases doubled. Lyme borreliosis accounted for 82% of all reported tick-borne diseases, and the incidence of anaplasmosis and ehrlichiosis increased yearly [4].

Vector-borne disease epidemiology is complex because of environmental influence on vectors [5]. The dynamics of vector-borne pathogen transmission are determined by the interactions between pathogen, vector, reservoir, host, and environmental factors [4, 5]. Furthermore, some arthropods are competent vectors for transmission of more than one pathogen [6]. Most vector-borne pathogens are zoonotic. Arthropod vectors can bridge the gap between animals and humans that do not ordinarily interact [4]. Vector-borne zoonoses, often having wild animal reservoirs, can be difficult or impossible to eradicate [4].

Tick-borne pathogens rarely cause sudden epidemics in people for several reasons. Tick mobility is limited to the movement of its animal hosts [7]. Humans are mostly incidental hosts. Additionally, the prolonged life cycle and widely separated blood meals of ticks limit their ability for rapid or wide-spread transmission of disease pathogens [4].

In addition to wildlife reservoir, pet animals have also been implicated in the transmission of zoonotic agents to humans. Pets may come in close contact with wild animals or vectors that have previously been exposed to reservoir animals [5]. As an example, dogs are competent reservoir hosts for several zoonotic vector-borne bacteria and protozoa and can be an important source of nutrition for many blood-sucking arthropods like fleas, mosquitoes, sand flies, and ticks [6, 8, 9]. This suggests that household pets may offer a relatively accessible sentinel for vector-borne zoonotic diseases.

Canine vector-borne diseases (CVBDs) are caused by widely distributed and various arthropod-borne pathogens [10]. Anaplasmosis and ehrlichiosis are emerging bacterial diseases that affect dogs and humans in North America and other parts of the world [11, 12]. *Anaplasma phagocytophilum* (*A. phagocytophilum*) is a causative agent of granulocytotropic anaplasmosis and is transmitted by *Ixodes* species ticks (e.g., the blacklegged tick, *Ixodes scapularis* (*I. scapularis*)) [13–15]. *Anaplasma platys* (*A. platys*), which is putatively transmitted by the brown dog tick, *Rhipicephalus sanguineu*s (*R. sanguineus*), causes canine infectious thrombocytopenia and was recently implicated in human disease cases [16–20]. *Ehrlichia canis* (*E. canis*) and *Ehrlichia ewingii* (*E. ewingii*), the agents of ehrlichiosis, are transmitted by *R. sanguineus* and the lone star tick, *Amblyoma americanum* (*A. americanum*), respectively [21, 22]. Importantly, dogs are considered natural hosts for *A. platys*, *E. canis*, and potentially *E. ewingii* [23, 24] and can remain infected for many months or years. In 2014, an infection with *A. platys*, *E. ewingii*, and *Ehrlichia chaffeenis* (*E. chaffeenis*) was described in a dog and two family members providing molecular evidence of persistent infection in humans [18]. Subsequently, a human chronic infection with *E. chaffeensis* was reported in 2015 [25]. The bacterial infections may develop into chronic debilitating diseases [21, 23, 26]. Dogs can also become persistently infected with *Borrelia burgdorferi* (*B. burgdorferi*) via exposure to *Ixodes* species ticks. In North America, human Lyme disease and canine borreliosis is caused by *B. burgdorferi* sensu stricto, which is transmitted by *I. scapularis* and *Ixodes pacificus* [27]. Finally, heartworm is one of the most important helminthic diseases of canids in North America and its causative agent is the mosquito-transmitted nematode, *Dirofilaria immitis* (*D. immitis*) (Leidy 1856) [28–30] *D. immitis* is responsible for canine cardiopulmonary dirofilariasis and human pulmonary dirofilariasis [31]. This zoonotic parasite is mainly located in temperate, tropical, and subtropical areas of the world [31–34]. Human cases have been reported mainly in areas of high canine prevalence, highlighting the importance of heartworm testing and chemoprophylaxis in dogs to reduce transmission [35].

Despite that the above described agents of CVBDs can be life-threatening for pets and directly affect human health, studies on the seroprevalence of vector-borne pathogens in animals are quite limited [5]. Therefore, the objective of this study was to determine the serological prevalence of six vector-borne pathogens in dogs (*A. phagocytophilum, A. platys*, *B. burgdorferi, E. canis, E. ewingii,* and *D. immitis*) presented for elective surgery at a high volume spay and neuter practice in the South Central region of Texas (US).

## Results

### Study animals

During the study period, 418 presumed healthy dogs were identified as serologically tested for the presence of antibodies to bacterial pathogens of anaplasmosis (*A. phagocytophilum* and *A. platys*), ehrlichiosis (*E. canis* and *E. ewingii*), and borreliosis (*B. burgdorferi*); and the antigen of heartworm (*D. immitis*). The study dogs included 192 (46%) males and 226 (54%) females. Their reported ages varied from 6 months to 18 years with the median age being 4 years. The data represented a total of 60 general breed types reported by owner/agents: Terrier mix (*n* = 51), Chihuahua (*n* = 50), Labrador Retriever (*n* = 41), Pit Bull (*n* = 25), Dachshund (*n* = 22), Shepherd mix (*n* = 17), Boxer (*n* = 13), Shih Tzu (*n* = 13), Chihuahua/Dachshund mix (*n* = 11), Great Pyrenees (*n* = 11), Australian Cattle Dog (*n* = 8), German Shepherd (n = 8), Poodle (*n* = 7), Schnauzer (n = 7), Beagle (*n* = 6), Border Collie (*n* = 6), Great Dane (*n* = 6), Husky (*n* = 6), Mastiff (*n* = 6), Miniature Pinscher (*n* = 6), Yorkshire Terrier (*n* = 6), Australian Shepherd (*n* = 5), Maltese (*n* = 5), Wirehaired Terrier (*n* = 5), Doberman Pinscher (*n* = 4), Heeler (*n* = 4), Hound (*n* = 4), Jack Russel Terrier (*n* = 4), Belgian Malinois (*n* = 3), Bull Dog (*n* = 3), Cairn Terrier (*n* = 3), Catahoula Leopard Dog (*n* = 3), Golden Retriever (*n* = 3), Papillon (*n* = 3), Retriever mix (*n* = 3), Shar Pei (*n* = 3), Spaniel (*n* = 3), Welsh Corgi (*n* = 3), Bassett Hound (*n* = 2), Black Mouth Cur (*n* = 2), Chow Chow (*n* = 2), Coonhound (*n* = 2), Lhasa Apso (*n* = 2), Pekingese (*n* = 2), Pointer (*n* = 2), Pomeranian (*n* = 2), Rottweiler (*n* = 2), American Eskimo Dog (*n* = 1), Basenji (*n* = 1), Bichon Frise (*n* = 1), Blue Lacy (*n* = 1), Boston Terrier (*n* = 1), Brussels Griffon (*n* = 1), Chinese Crested (*n* = 1), Cocker Spaniel (*n* = 1), Manchester Terrier (*n* = 1), Pug (*n* = 1), Rhodesian Ridgeback (*n* = 1), Weimaraner (*n* = 1), and West Highland Terrier (*n* = 1). Based upon the information provided by the owners or agents, 62 (14.8%) dogs were privately-owned and the other 356 (85.2%) dogs were rescued or under the possession of community animal control facilities. Of the privately-owned animals with pre-existing medical records, 21 (33.9%) were being administered flea and/or heartworm prophylaxis at the time of blood collection.

### Serology

A summary of SNAP 4Dx Plus (IDEXX Laboratories Inc., Westbrook, ME, US) (SNAP) test results is provided in Table 1. The combined data showed that 20.3% of the dogs (85 out of 418) were exposed to or infected with at least one of the 6 pathogens. There were no significant differences associated with sex or age between the dogs with positive and negative test results. However, the dogs that tested positive for any of the 6 pathogens (the median body weight of 19.0 kgs, range 2.5 to 56.0 kgs) were significantly heavier than the serologically negative dogs (median 10.9 kgs, range 1.4 to 55.5 kgs; *P* = 0.01).

**Table 1:** Summary of vector-borne disease serological test results for canine patients admitted to a high volume spay and neuter practice in the South Central region of Texas over 2014–2019

Categories	Number (%) of positive dogs via SNAP tests by year						Total
Year	2014	2015	2016	2017	2018	2019	
*An* ab^a^	2 (3.7)	0 (0)	0 (0)	6 (6.5)	5 (4.9)	5 (3.8)	18 (4.3)
*Bb* ab^a^	0 (0)	0 (0)	1 (4.5)	0 (0)	0 (0)	0 (0)	1 (0.2)
*Eh* ab^a^	7 *(13.0)*	0 (0)	2 (9.1)	11 (11.8)	6 (5.8)	9 (6.8)	35 (8.4)
*Di* ag^a^	5 (9.3)	3 (21.4)	3 (13.6)	8 (8.6)	12 (11.7)	18 (13.6)	49 (11.7)
Total test-positive dogs^b^	11 (20.4)	3 (21.4)	4 (18.2)	20 (21.5)	19 (18.4)	28 (21.2)	85 (20.3)
Total test-negative dogs^c^	43 (79.6)	11 (78.6)	18 (81.8)	73 (78.5)	84 (81.6)	104 (78.8)	333 (79.7)
Total dogs tested	54	14	22	93	103	132	418

^a^*An* ab, anti-*Anaplasma phagocytophilum* and/or *Anaplasma platys* antibodies; *Bb* ab, anti-*Borrelia burgdorferi* antibodies; *Eh* ab, anti-*Ehrlichia canis* and/or *Ehrlichia ewingii* antibodies; *Di* ag, *Dirofilaria immitis* antigen

^b^Number of positive dogs by any of the four SNAP test results

^c^Number of negative dogs by all four SNAP test results

The results showed that 4.3% of the dogs (11 females and 7 males) tested positive for antibodies to *A. phagocytophilum* and/or *A. platys* (Table 1). The median age was 4 years (range 10 months to 8 years) and 3 dogs were reported under 2 years of age. The median weight was 14.4 kgs (range 2.5 to 34.1 kgs). No significant differences associated with age or weight of the *Anaplasma*-positive dogs were identified when compared to the dogs that tested negative by the SNAP test.

There were 8.4% of the dogs (15 females and 20 males) that tested positive for anti-*Ehrlichia* antibodies (Table 1). The median age and weight were 4.5 years (range 10 months to 10 years) and 15.1 kgs (range 2.5 to 33 kgs), respectively. Four dogs were reported to be under 2 years of age. There were no significant differences in age or weight between the *Ehrlichia*-positive and serologically negative dogs. Two of the 35 dogs were retested a month later, and their blood samples remained positive (data not shown).

Of the 11.7% of the dogs (25 females and 24 males) that tested positive for canine heartworm, the median age of 5 years (range 6 months to 11 years) was significantly higher than the respective ages of the dogs that tested negative for any of the other 5 pathogens (*P* < 0.01). Only 2 dogs were reported to be under 2 years of age. The median weight for the heartworm antigen-positive dogs was 20.2 kgs (range 2.7 to 56.0 kgs), which was heavier than the dogs that were negative for any of the other pathogens tested (*P* < 0.05).

In contrast to the above test results, only one male dog out of 418 (0.2%) tested positive for antibodies to *B. burgdorferi* (Table 1). Exposure to or infection with 2 or more pathogens (referred to here as co-infection) was detected in 3.8% out of 418 dogs (7 females and 9 males). Specifically, 8 dogs were serologically positive for *Anaplasma* and *Ehrlichia*, and 4 dogs tested positive for *Ehrlichia* and heartworm. One dog tested positive for *Anaplasma* and heartworm, and another dog was seropositive for *B. burgdorferi* and *Ehrlichia*. Lastly, two dogs tested positive for *Anaplasma*, *Ehrlichia*, and heartworm. The median age for the co-infected animals was 4 years (range 2 to 6 years). The dogs’ median weight was 9 kgs (range 2.5 to 32 kgs).

### Estimated true prevalence

Based on the sensitivity and specificity of the SNAP test, the estimated true prevalence (95% CI) was calculated for *Ehrlichia*, 3.98% (1.50 to 7.28% (Lower, Upper 95% CI)) and *D. immitis*, 11.21% (8.42 to 14.71%)*.* Given the low number of dogs that tested positive for *Anaplasma* (*n* = 18) or *B. burgdorferi* (*n* = 1), it was not possible to accurately assess the true prevalence for these two pathogens.

## Discussion

Dogs are considered not only biological hosts for most canine vector-borne diseases, but also important environmental sentinels for determining the frequency and distribution of infected vector populations [36–39]. Moreover, recently, dogs facilitated the efforts to better understand the potential public health implications of various canine vector-borne disease pathogens [6]. Given their sentinel role and the scarcity of seroprevalence data in dog populations, the objective of this study was to use a convenience sample of dogs presenting for elective surgical neutering at a high volume surgical practice to identify the serological prevalence of *A. phagocytophilum, A. platys*, *B. burgdorferi, E. canis, E. ewingii,* and *D. immitis* in dogs from the Greater San Antonio area. Based upon the information provided by the owners or agents, 62 (14.8%) dogs in this study were privately-owned and the other 356 (85.2%) dogs were rescued or under the possession of community animal control facilities. The City of San Antonio’s Animal Care Services conducted a study in 2019 to determine the total number of unrestrained dogs that were owned “roaming” and unowned “stray” animals. The results of their study predicted that 87.2–96.5% (CI 95%) of all unrestrained dogs were likely owned “roaming” dogs [40]. This suggests that, in addition to the known privately-owned dogs, a high percentage of the present study’s rescued and stray dogs were likely previously owned and lived in close contact with humans.

The present study showed that the highest apparent seroprevalence rate combined for the 6-year period was 11.7% for *D. immitis*. The heartworm antigen was also detected in almost half of the co-infected dogs. Such a high level of heartworm seroprevalence among the dog population in and around the city of San Antonio was not surprising. Furthermore, it is possible that the actual prevalence could be even higher as antigen blocking due to immune complex formation within the host may be a cause for false negative serologic test results [41, 42]. Historically, the counties of the studied area have had endemic foci, and the seroprevalence rates were shown to vary from 6.1 to 20.0% [29, 43]. For general comparison, the serological survey previously conducted in the continental US demonstrated that five southeastern states, namely Arkansas (6.8%), Louisiana (6.0%), Mississippi (7.4%), South Carolina (5.7%), and Texas (5.5%) had the highest seroprevalence rates of *D. immitis* antigen-positive dogs [29]. To contrast the regional prevalence for the Southeast (2.9–3.9%), the respective rates for the states of the Northeast, Midwest, and West ranged from 0.4 to 1.2% [29, 42].

Similar to the heartworm rate, the apparent seroprevalence of *E. canis* and/or *E. ewingii* was high (8.4%); however, this finding was quite unexpected. Previous studies demonstrated that, in Texas, the mean seroprevalence varied from 0.8 to 3.6% and the rates for the counties of the Greater San Antonio area did not exceed 2.0% [29, 43, 44]. For comparison, the mean seroprevalence of the seropositive dogs across 48 states was 0.6% with the highest rates being observed in Arkansas (3.9%), Oklahoma (3.8%), Arizona (3.2%), Mississippi (3.1%), Tennessee (2.3%), Kansas (2.2%), North Carolina (2.1%), Georgia (1.9%), Missouri (1.9%), and Virginia (1.8%) [29]. Such an aberrantly high seroprevalence observed in this study could be potentially explained by two factors. Unlike the previous study where the test detected only antibodies to *E. canis* [29], the SNAP system used in the current work had the capacity to identify antibodies against an additional *Ehrlichia* species, *E. ewingii* [45]. Moreover, most of the dogs in this study (85.2%) were from rescue groups or shelters. Thus, the fact that stray, rescued, or surrendered dogs were likely more frequently exposed to ticks (*A. americanum* and *R. sanguineus*) and devoid of any tick preventive products, could account for the high prevalence of anti-*Ehrlichia* antibodies in the studied dog population.

Compared to the heartworm and *Ehrlichia* seroprevalence rates, antibodies to *A. phagocytophilum* and/or *A. platys* were detected in much fewer dogs of the examined cohort (4.3%). This seroprevalence rate was approximately several fold higher than the respective rates previously identified in Texas (0.6%) and the other southeastern states (0.5–1.2%) [29]. However, in 2019, Hodo et al. reported approximately 16% prevalence of *Anaplasma* spp. from a shelter in San Antonio proper [43]. The *Anaplasma* seroreactive dogs of this study were most likely exposed to *A. platys* rather than *A. phagocytophilum*, particularly as 8 dogs were *Ehrlichia* and *Anaplasma* seroreactive. Also, since 92% of the seropositive dogs were strays and maintained in shelters, *R. sanguineus* infestation (kennel tick) would be likely responsible for the transmission [45–49].

Contrary to the above results, the seroprevalence of *B. burgdorferi* identified in the current and previous studies is almost identical (0.2–0.3%) and is well consistent with an overall low regional rate for the Southeast of the US (1.0%) [27, 29]. Of note, Texas belongs to the low incidence state category with the mean annual rate of confirmed human Lyme borreliosis being 0.2 cases per 100,000 population [50]. Interestingly, this incidence coincided with the seroprevalence rate of *B. burgdorferi* among dog populations examined in Texas by this and previous studies [27, 29]. It also should be mentioned that exposure to tick-borne relapsing fever *Borrelia* occurs in Texas; however, based upon a recent publication the *B. burgdorferi* C6 peptide-based SNAP test did not result in a false positive in experimentally infected dogs [51]. Also, in this study a travel history could not be established for the *B. burgdorferi*-positive dog. Lastly, the study showed that almost 4% of the dogs were co-exposed or co-infected with 2–3 pathogens, indicating that the animals were exposed to different vectors during their lifetime [8, 9, 52].

It is also important to note that this study had several limitations. First, the limited number of the examined animals could not fully reflect serological status of the entire dog population in the studied region. Additionally, the animals selected for sample collection likely represented a skewed cohort as there were no standardized inclusion criteria. Finally, serological testing alone could underestimate the actual disease prevalence. Previous heartworm studies reported that commercial serological kits may have low sensitivity when there are low parasite burdens, prepatent infections, infections by aging adult female worms with impaired fertility, infections with male-only worms, or when the presence of microfilariae persists for 1–3 years after the death of adult females [31, 53]. Thus, it is probable that the actual number of vector-borne disease cases in and around the Greater San Antonio area exceed those projected by this study.

## Conclusions

Serology continues to be an important epidemiological modality to estimate the prevalence of zoonotic vector-borne diseases among dogs. The presented data may be useful to estimate the respective risks to human populations. Opportunistic testing of dogs presenting for elective surgical procedures may offer an effective way of assessing prevalence and/or risk factors for vector-borne diseases common to dogs and humans within a geographical region of interest.

## Methods

**Fig. 1:**
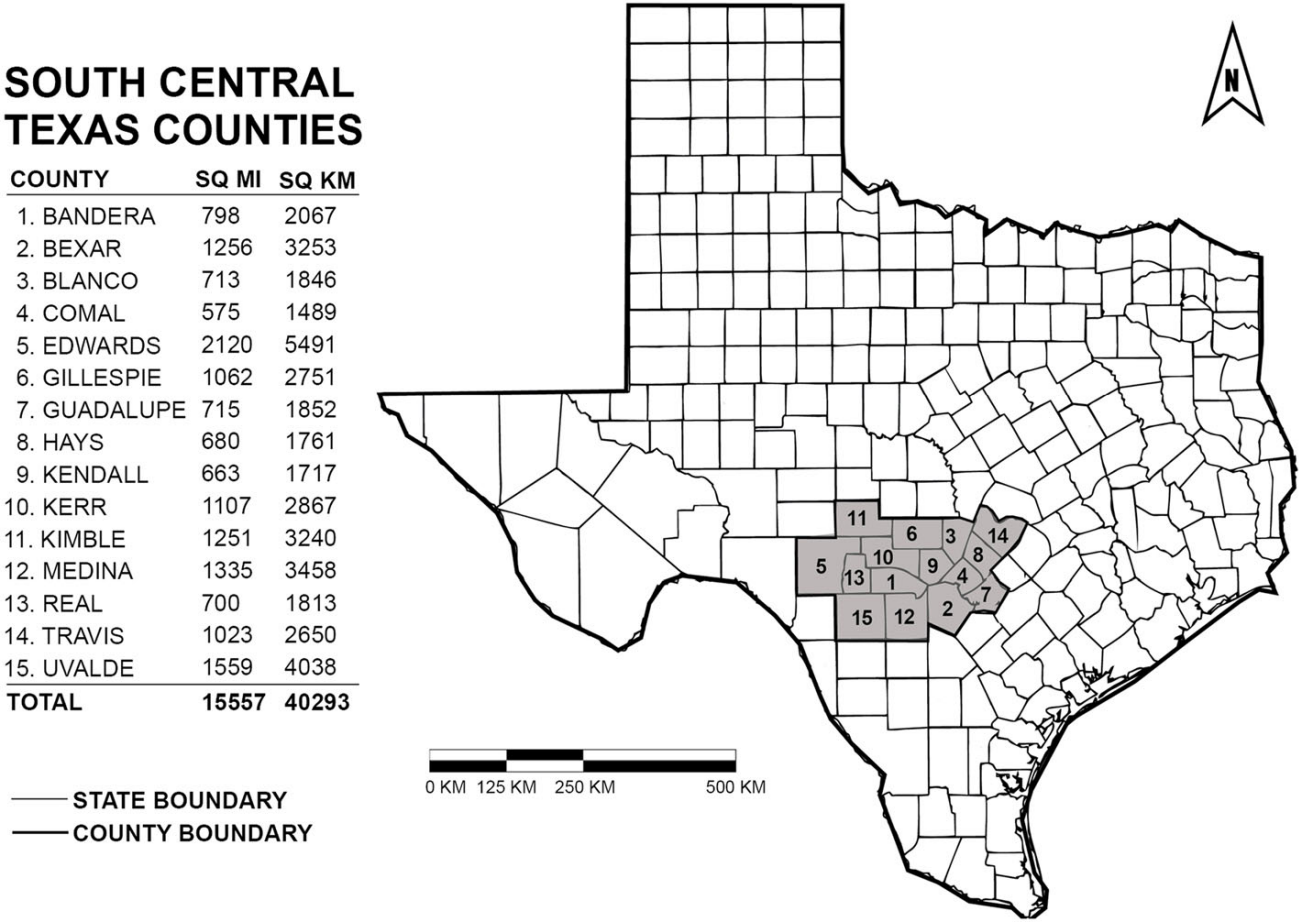
The South Central region of Texas (the Greater San Antonio area). The map of the state of Texas (US) depicts all 15 counties from which the studied dogs were originated. The overall area of these counties is 15,557 mile^2^ (40,292 km^2^). The counties included: Bandera, Bexar, Blanco, Comal, Edwards, Gillespie, Guadalupe, Hays, Kendall, Kerr, Kimble, Medina, Real, Travis, and Uvalde. SQ MI and SQ KM denote square miles and square kilometers, respectively. The map was generated by using the 2019 Adobe Creative Cloud suite (Illustrator, Photoshop, and InDesign; www.adobe.com)

Sera from dogs of different breeds were collected for serological testing as part of routine diagnostic service, and the data analysis was performed retrospectively, therefore no consent to participate in this study was required. Dogs’ sera were screened using commercial ELISA-based test, SNAP 4Dx Plus, which detected antibodies to five bacterial pathogens, *A. phagocytophilum, A. platys*, *B. burgdorferi, E. canis, E. ewingii,* and an antigen of canine heartworm, *D. immitis* [45, 48, 49]. Sensitivity (95% CI) and specificity (95% CI) for the SNAP test as reported by the manufacturer were: 99.0% (94.3–99.9%) and 99.3% (97.4–99.9%) for heartworm; 90.3% (85.8–93.7%) and 94.3% (90.7–96.7%) for *Anaplasma*; 97.1% (94.0–98.8%) and 95.3% (92.7–97.2%) for *Ehrlichia*; and 94.1% (88.3–97.6%) and 96.2% (92.9–98.3%) for *B. burgdorferi* [54]. Electronic medical records of dogs presented for elective ovariohysterectomy or castration at a high volume spay and neuter clinic in Boerne, Texas from June 2014 through December 2019 inclusive, were reviewed for the vector-borne disease serological test results. Included dogs belonged to a variety of private owners, rescue groups, and community animal control facilities. The studied dogs came from a 15-county region of the South Central Texas (the Greater San Antonio area) representing a geographical area of 15,557 mile^2^ (40,292 km^2^) (Fig. 1).

Statistical analysis was performed using Microsoft Excel 2016 and statistical software R 3.5.0 (R Core Team, 2018) [55]. For each examined variable, descriptive statistics were computed. Since the ages and body weights of dogs testing negative and positive for the assessed vector-borne diseases were not normally distributed, differences were determined using the non-parametric Mann-Whitney test. Pearson chi-square test was used to detect the sex difference between negative and positive groups. Statistical significance was set at *P* < 0.05. Estimated true prevalence (95% CI) calculations were performed using Epitools Epidemiological Calculators [56].

## Data Availability

The datasets used and/or analysed during the current study are available from the corresponding author on reasonable request.

## Abbreviations

CVBDs: Canine vector-borne diseases
CI: Confidence interval
ELISA: Enzyme-linked immunosorbent assay
SNAP: SNAP 4Dx Plus test
US: United States of America
